# Subpixel Edge Localization via Ruled-Sigmoid Surfaces and Its Application for Precision Analysis of Cycloidal Gear Profiles

**DOI:** 10.3390/s26134193

**Published:** 2026-07-02

**Authors:** Jing Zhang, Po Du, Wenzhen Zhao, Wenhui Zhao

**Affiliations:** School of Mechanical Engineering, Shenyang University of Technology, Shenyang 110870, China; dupo@sut.edu.cn (P.D.); zhaowenzhensut@163.com (W.Z.); zhaowenhui@sut.edu.cn (W.Z.)

**Keywords:** visual measurement, cycloidal gear, subpixel, sigmoid, edge localization

## Abstract

To overcome the limitations of single-dimensional data and low efficiency in traditional cycloidal gear inspection, a comprehensive machine vision-based method was proposed. A high-precision vision platform was established, and a Sigmoid surface-based edge detection algorithm was employed for sub-pixel edge localization. Logarithmic transformation combined with light intensity compensation was applied to correct saturation-induced errors. The pixel equivalent and compensation coefficient were systematically calibrated using a dot-matrix plate and gauge blocks. A sub-pixel tooth profile model in the physical coordinate system was reconstructed through pixel equivalent calibration, dynamic light intensity compensation, and multi-coordinate transformation. Comparative tests against a coordinate measuring machine (CMM) verified that the point-to-point deviation between the two measurement systems was within 10 μm (maximum 11.62 μm). The inherent tooth profile deviation of the tested cycloidal gears, which reflects the machining quality of workpieces, ranged from 24 μm to 37 μm. Multiple repeated tests prove that the system achieves a repeat positioning accuracy of 0.8 μm. Based on the measurement characteristics, a hybrid analytical method integrating Cartesian and polar coordinate systems was developed, enabling the simultaneous evaluation of critical geometric tolerances, such as the diameters of the center hole and crankshaft hole. The full inspection cycle for cycloidal gears was reduced to 13 s, which demonstrates a substantial efficiency improvement over traditional methods.

## 1. Introduction

Cycloidal gears are the core transmission components of RV reducers. The transmission efficiency, motion smoothness, noise level, and service life of the reducer are directly influenced by the tooth profile accuracy. With the growing demand for high-precision transmission in industrial robots, precision machine tools, and aerospace, precise inspection of cycloidal gears has become a key technical link for ensuring the performance and reliability of high-end equipment [1,2,3]. An RV reducer consists of a first-stage involute cylindrical gear planetary mechanism and a second-stage cycloidal pinwheel planetary mechanism. The cycloidal pinwheel mechanism is directly connected to the output shaft, and its tooth profile machining error has a decisive influence on the motion accuracy and repeat positioning accuracy of the reducer [4]. Therefore, high-precision and high-efficiency measurement of cycloidal gear tooth profile deviations is of great significance for improving the manufacturing level of domestic RV reducers [5].

Traditional measurement methods for cycloidal gears can be divided into contact and non-contact approaches [6]. Contact measurement, represented by coordinate measuring machines (CMMs), offers high absolute accuracy but suffers from low efficiency due to point-by-point scanning, potential minor deformations caused by the measurement force, and difficulty in achieving rapid whole-profile inspection and online evaluation [7,8]. Non-contact methods include laser measurement and vision-based measurement. Laser measurement, based on triangulation ranging, enables fast scanning; however, its accuracy is often lower than that of CMMs due to surface reflection characteristics and sensor inclination errors [9]. In contrast, vision-based measurement has gradually become an important means for inspecting geometric parameters of precision parts because of its high efficiency, full-field capability, and non-contact nature [10,11]. Yu et al. constructed a hybrid laser-vision system to realize full tooth profile reconstruction and geometric error evaluation for small-modulus gears. Nevertheless, the system relies on expensive line laser hardware and complex multi-sensor calibration procedures, which increases the equipment cost and computation burden, and is unsuitable for low-cost full-field visual inspection platforms only equipped with telecentric lenses [12].

In vision-based measurement, the accuracy of edge positioning directly determines the accuracy of dimensional measurement. Pixel-level edge positioning can no longer meet the micron- or even sub-micron-level precision requirements, making subpixel edge positioning a key technology for improving visual measurement accuracy [13,14]. Current mainstream subpixel edge positioning algorithms fall into three categories: interpolation methods, fitting methods, and moment methods. Interpolation methods (e.g., bicubic interpolation, cubic spline interpolation) have low computational complexity but limited accuracy and high sensitivity to noise [15]. Moment methods (e.g., grayscale moment, spatial moment, Zernike moment) offer good noise immunity and rotational invariance, with the Zernike moment method being the most widely used [16,17]. Song and He [18] adopted Zernike moment subpixel extraction to measure elliptical gear contours, but the algorithm involves heavy matrix computation and only adapts to smooth elliptical tooth surfaces without sharp curvature mutations, which cannot be applied to cycloidal gears with drastic changes at the addenda and dedenda. Fitting methods achieve subpixel positioning by constructing a grayscale distribution model and feature good noise robustness and high accuracy [19]. Among these, the Gaussian integral surface model is a commonly used edge grayscale distribution model, but its inverse function requires table lookup, making the computation complex and difficult to meet real-time requirements [20].

The sigmoid function has been recognized as a close approximation to the Gaussian integral curve in the edge transition region, while offering the distinct advantage of an analytically solvable inverse function. The shape similarity between the logistic sigmoid function and the standard normal cumulative distribution function (CDF) has been quantitatively validated in recent literature [21,22]. Froelich (2025) introduced the prime density function, a logistic-cubic closed-form approximation to the normal CDF, and reported a maximum absolute error below 1.7 × 10^−4^ when fitting the standard normal CDF with an optimized logistic-based formulation [21]. This quantitative evidence confirms the high degree of shape correspondence between the two functions in the mid-range transition region, establishing the sigmoid function as a theoretically sound substitute for the Gaussian integral model in edge modeling. However, when directly applied to complex surface fitting, the sigmoid model remains computationally demanding, motivating the further simplification proposed in this work.

To address the above problems, this paper proposes a subpixel edge localization method based on the ruled-sigmoid surface model [23,24]. Through a mathematical transformation, the proposed method converts the nonlinear sigmoid surface into a linear (ruled) surface model, which significantly simplifies the solution process while preserving the advantages of the sigmoid function. A light-intensity compensation coefficient is introduced to effectively suppress edge offset errors caused by image oversaturation or undersaturation [25]. This coefficient is calibrated using gauge blocks, as detailed in Section 5. Based on this method, a vision measurement system integrating a high-resolution CMOS camera, dual telecentric lenses, and an LED backlight is built. A coordinate transformation model that accounts for gear positioning errors is established to enable high-precision measurement of the tooth profile deviation and pitch deviation of cycloidal gears [26,27,28,29].

This paper presents a subpixel edge localization method based on a ruled-sigmoid surface model. The key novelty is a mathematical transformation that converts the nonlinear surface into a linear ruled surface, which greatly simplifies the solution while retaining the benefits of the sigmoid function. Furthermore, a light-intensity compensation mechanism is integrated into the edge positioning model to suppress offset errors caused by saturation. Using these techniques, a composite error quantification model is established to characterize deviations of both tooth profile and pitch, and its accuracy and efficiency are verified through experiments.

## 2. Cycloidal Gear Measurement System

### 2.1. Hardware Subsystem

**Figure 1:**
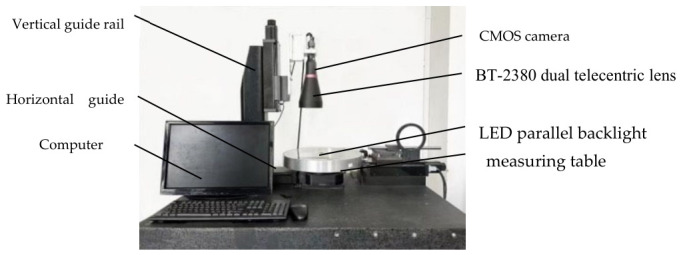
Visual measurement system.

The proposed cycloidal gear measurement system consists of hardware and software subsystems. The hardware subsystem is further divided into a mechanical structure and an image acquisition module. The mechanical structure, as shown in Figure 1, comprises a granite platform, a vertical column, two mutually perpendicular guide rails, and a stepping motor. A perpendicular configuration of the two guide rails is adopted to ensure high stability and measurement accuracy. The industrial camera is mounted on the guide rails: the vertical rail (object distance adjustment mechanism) employs a ball screw to adjust the working distance of the camera and lens, enabling sharp focusing; the horizontal rail adjusts the lateral field of view to ensure that the entire cycloidal gear remains within the camera’s coverage. The image acquisition module consists of an MV-HS2000GM industrial CMOS camera (resolution: 5472 × 3648 pixels, pixel size: 2.4 μm × 2.4 μm), a BT-2380 dual telecentric lens (field of view: 80 mm × 60 mm), an LED parallel backlight, a working plane, and a computer. High-quality images are captured by the camera, lens, and light source operating in concert, and are then transmitted to the computer via a network-interface image acquisition card.

### 2.2. Software System

**Figure 2:**
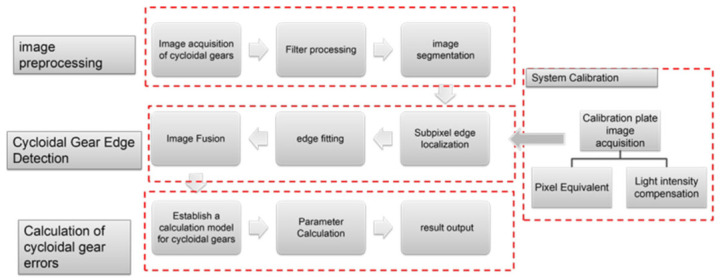
The block diagram of the software system.

The software subsystem consists of image preprocessing, system calibration, subpixel edge localization based on the ruled-sigmoid surface model, and cycloidal gear error calculation. The pixel equivalent and the light-intensity compensation coefficient are calibrated using gauge blocks. After the image of the cycloidal gear under test is acquired, the ruled-sigmoid surface model is used for subpixel edge localization, and then the light-intensity compensation coefficient is applied to correct the edge positioning errors caused by variations in image saturation. Based on the tooth profile equation of the cycloidal gear, computational models for pitch deviation and tooth profile deviation are established to enable error evaluation. Figure 2 shows the software block diagram of the vision measurement system.

### 2.3. Measurement Workflow

The measurement system operates according to the following steps:

1. Mechanical adjustment
  Adjust the object distance and the perpendicularity between the measurement plane and the camera to meet the experimental requirements.
2. Image acquisition
  Capture the cycloidal gear image using the image acquisition module and transfer it to the computer.
3. Software processing
  Process the acquired image using the ruled-sigmoid surface model to extract the subpixel edges and coordinate points of the cycloidal gear, and then calculate the gear errors (pitch deviation and tooth profile deviation).

## 3. Edge Positioning Algorithm

### 3.1. Construction of Ruled-Sigmoid Surfaces

In vision-based measurement, edge positioning is typically performed on a digital image grayscale matrix [30]. The grayscale values of the image are normalized. The row and column indices of each matrix element are taken as the y- and x-coordinates of the corresponding pixel, respectively, while the element value represents the grayscale intensity at that location. When such a matrix is interpreted as a surface with coordinates (x,y,z), it is referred to as a grayscale surface.

**Figure 3:**
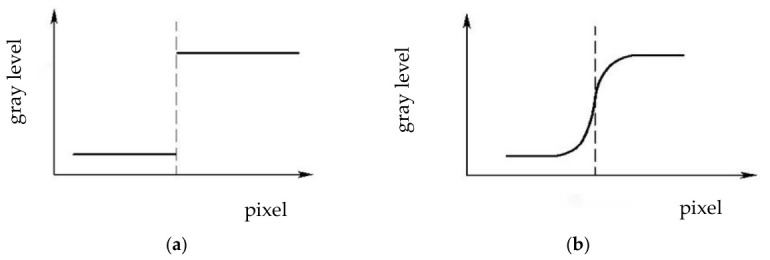
Ideal step edge and actual edge normal models. (**a**) Ideal step edge; (**b**) effective edge.

Ideally, the cross-sectional curve of a normal edge on a grayscale surface is a step function, as illustrated in Figure 3a. In practice, however, due to the point spread function of the lens, the actual edge is the convolution of the ideal step edge with the point spread function, which acts as a low-pass filter on the image. Consequently, some high-frequency information is lost, and the edge becomes blurred, resulting in a transition region of finite width between the background and the foreground, as shown in Figure 3b. The grayscale variation across this transition region can be described by a Gaussian integral curve [31].

The sigmoid function closely coincides with the Gaussian integral curve in the edge transition region and offers the advantage of an analytically solvable inverse function, thereby eliminating the need for table lookup required by Gaussian surface fitting [32]. Moreover, its functional form can adapt to the edge expression to be determined. Therefore, in this work, the sigmoid curve is adopted to replace the Gaussian integral curve for modeling the normal profile of a real edge. The edge grayscale surface is then represented by a sigmoid surface, which is generated by translating the sigmoid curve along the edge direction.

Although it is theoretically feasible to directly apply the sigmoid surface for subpixel edge localization, the resulting model is computationally complex and difficult to solve. To overcome this limitation, a mathematical transformation leveraging the properties of the sigmoid function is performed, leading to a subpixel edge localization method based on a linear (ruled) surface model, i.e., the ruled-sigmoid surface.

### 3.2. Subpixel Edge Localization Based on Ruled-Sigmoid Surface

Building upon the ruled-sigmoid surface model introduced in Section 3.1, this section presents a computationally efficient subpixel edge localization algorithm. The key idea is to exploit the analytical invertibility of the sigmoid function to transform the nonlinear grayscale surface into a linear form, enabling closed-form least-squares estimation of the subpixel edge parameters.

#### 3.2.1. Linearization of the Sigmoid Surface

Let the normalized grayscale value at a pixel $((x_{j},y_{j}))$ within the edge transition band be denoted by $(G(x_{j},y_{j}))$. According to the ruled-sigmoid model, the grayscale distribution along the edge normal direction follows a sigmoid function:

(1) $$G(x_{j},y_{j})=\frac{1}{1+\exp\left(-\frac{x_{j}-a_{i}-k_{i}\Delta y_{j}}{b_{i}}\right)},\;\Delta y_{j}=y_{j}-y_{i}$$

where $\left(a_{i}\right)$ is the subpixel edge position in the (x)-direction, $\left(b_{i}\;>\;0\right)$ is the slope coefficient (edge steepness), and $\left(k_{i}\right)$ is the local edge slope. This model assumes that within a small tangential window $\left(y\in (y_{i}-\delta y,y_{i}+\delta y)\right)$, the edge profile is invariant along the (y)-axis—a reasonable approximation for smoothly curved edges such as gear tooth profiles.

Taking the natural logarithm and applying the inverse sigmoid transformation yields a strictly linear relationship:

$$x_{j}=a_{i}+b_{i}\cdot L_{j}+k_{i}\Delta y_{j},\;\mathrm{where}\;L_{j}=\ln\frac{G(x_{j},y_{j})}{1-G(x_{j},y_{j})}.$$

Thus, for a given local segment (i), each pixel $((x_{j},y_{j},G_{j}))$ maps to a point $((L_{j},\Delta y_{j}))$ in a transformed space, and the parameters $((a_{i},b_{i},k_{i}))$ become the coefficients of a plane.

Affected by image saturation, edge positions usually deviate, and the precise boundaries obtained by edge extraction algorithms are often the equidistant lines of the actual boundaries [33]. By performing compensation and adjustment L=L+λ, the position of the calculated precise boundary is corrected to make it closer to the actual boundary and improve calculation accuracy. Through calibration experiments, the logarithmic grayscale compensation value λ is determined, and the calibrated parameter λ serves as the light intensity compensation coefficient of the system. The specific calibration process is described in Section 5.2.

#### 3.2.2. Linear Least-Squares Parameter Estimation

Given (m) pixels within the transition region of the (i)-th edge segment, an overdetermined linear system was constructed:

(2) $$\left[\begin{array}{c} x_{1}\\ x_{2}\\ \vdots \\ x_{m} \end{array}\right]=\left[\begin{array}{ccc} 1 & L_{1} & \Delta y_{1}\\ 1 & L_{2} & \Delta y_{2}\\ \vdots & \vdots & \vdots \\ 1 & L_{m} & \Delta y_{m} \end{array}\right]\left[\begin{array}{c} a_{i}\\ b_{i}\\ k_{i} \end{array}\right]+\varepsilon ,$$

where (ε) represents residuals. The least-squares solution minimizes $\left(\sum_{j=1}^{m}(x_{j}-a_{i}-b_{i}L_{j}-k_{i}\Delta y_{j}{)}^{2}\right)$. Setting the partial derivatives to zero and rearranging yields the following linear system for the parameters:

(3) $$\left[\begin{array}{c} {\bar{x}}_{i}\\ {\bar{\mathrm{xL}}}_{i}\\ {\bar{x\Delta y}}_{i} \end{array}\right]=\left[\begin{array}{ccc} 1 & {\bar{L}}_{i} & {\bar{\Delta y}}_{i}\\ {\bar{L}}_{i} & {\bar{L^{2}}}_{i} & {\bar{\Delta \mathrm{yL}}}_{i}\\ {\bar{\Delta y}}_{i} & {\bar{\Delta \mathrm{yL}}}_{i} & {\bar{\Delta y^{2}}}_{i} \end{array}\right]\left[\begin{array}{c} a_{i}\\ b_{i}\\ k_{i} \end{array}\right],$$

where the overline denotes the mean over the (m) pixels, e.g., $\left({\bar{x}}_{i}=\frac{1}{m}\sum_{j=1}^{m}x_{j}\right)$. The parameters are obtained by solving this (3 × 3) linear system via matrix inversion or Gaussian elimination. The subpixel edge point is then $((a_{i},y_{i}))\;\mathrm{when}\;\left(\right|k_{i}\left|<1\right)$; otherwise, the roles of (x) and (y) are swapped and the calculation is repeated.

#### 3.2.3. Algorithmic Implementation

The proposed ruled-sigmoid edge localization algorithm proceeds as follows:

1. Preprocessing: Apply Gaussian filtering to suppress noise, then use a double-threshold method to extract the edge transition band (pixel-level region of interest).
2. Segmentation: Divide the edge band into overlapping segments of length (2δy) (typically (5)–(7) pixels) along the (y)-direction with a step of $\left(l_{y}\right)$.
3. Parameter estimation: For each segment, compute $\left(L_{j}\right)$ from the normalized grayscale values, build the linear system, and solve for $((a_{i},b_{i},k_{i}))$.
4. Subpixel edge assembly: Collect all $((a_{i},y_{i}))$ points. Optionally, apply a B-spline or moving average filter to remove outliers.
5. Coordinate transformation: Convert subpixel coordinates from the pixel coordinate system to the physical coordinate system using pre-calibrated pixel equivalent and distortion parameters. All segmentation and threshold parameters adopted in this algorithm are fixed through repeated pre-experiments to guarantee stable edge extraction under regular lighting conditions.

The entire algorithm is non-iterative and requires only basic linear algebra, making it suitable for real-time inspection. Experimental results (see Section 5) demonstrate a localization repeatability of better than 0.1 pixel and a total measurement cycle of approximately 13 s for a complete cycloidal gear.

### 3.3. Performance Verification and Comparison of Subpixel Algorithms

To quantitatively evaluate the positioning accuracy and measurement stability of the proposed ruled-sigmoid subpixel edge localization algorithm, a standard gauge block with a nominal length of 20 mm was adopted as the test specimen. The high-precision straight edge of the gauge block provides an ideal linear reference, which effectively eliminates interference from complex surface topography and assembly deviation. Comparative experiments were conducted with two widely used classic subpixel algorithms: the Gaussian integral surface fitting algorithm and the Zernike moment algorithm. All experiments were performed under identical imaging system parameters and camera calibration conditions to guarantee the fairness and comparability of the test results.

The quantitative performance comparison results are summarized in Table 1.

As shown in the table, the proposed ruled-sigmoid algorithm achieved optimal comprehensive performance among all tested algorithms. It obtained the minimum average positioning error of 0.08 pixel and the lowest root mean square error (RMSE) of 0.09 pixel. Compared with the Gaussian integral surface method and the Zernike moment algorithm, the proposed algorithm reduced the RMSE by approximately 47% and 65%, respectively. Meanwhile, the proposed algorithm exhibited a measurement repeatability of 0.01 pixel, which is far superior to the two contrast algorithms, fully verifying its excellent measurement stability and anti-interference ability. Benefiting from the linearization transformation strategy and efficient least-squares solution framework, the single-frame computational time of the proposed algorithm was reduced to 12.7 ms, which is approximately 56% lower than that of the Gaussian integral surface method, meeting the real-time requirements of practical industrial measurement scenarios.

## 4. Error Measurement Model for Cycloidal Gears

### 4.1. Pitch Deviation Measurement

The tooth pitch accuracy evaluation items included individual tooth pitch deviations affecting the operational stability of cycloidal gears, cumulative tooth pitch deviations, and cumulative total tooth pitch deviations influencing transmission motion accuracy [33].

According to the maximum and minimum polar radii of the cycloidal gear tooth profile, the polar radius $\left(\rho_{0}\right)$ of the circle close to the middle of the tooth height is determined. The right intersection point between the circle of radius $\left(\rho_{0}\right)$ and the actual tooth profile is then located. Because the edge coordinate points obtained by vision measurement are densely sampled, it can be assumed that $((\theta_{i-1},\rho_{i-1}))$, $((\theta_{0},\rho_{0})),$ and $((\theta_{i+1},\rho_{i+1}))$ lie on a straight line. Hence, the abscissa $\left(\theta_{0}\right)$ of the right intersection point can be obtained using Equation (4). The individual pitch deviation $\left(f_{\mathrm{pti}}\right)$ is then calculated according to Equations (5) and (6), where Equation (6) computes the difference between the angle of two adjacent homologous tooth flanks and the theoretical angle. The cumulative pitch deviation and the total cumulative pitch deviation are obtained from Equation (7) and Equation (8), respectively.

(4) $$\frac{\theta_{0}-\theta_{i-1}}{\theta_{i+1}-\theta_{i-1}}=\frac{\rho_{0}-\rho_{i-1}}{\rho_{i+1}-\rho_{i-1}},$$

where $(\rho_{0}\;=\;(\rho_{\max}+\rho_{\min})/2)$.

(5) Δφ(i)=φ(i)−φ(i−1),

(6) $$f_{\mathrm{pti}}=\left(\Delta \varphi (i)-\frac{2\pi }{z_{c}}\right)\rho_{0},$$

The cumulative pitch deviation from the first tooth to the k-th tooth is

(7) $$F_{\mathrm{pk}}=\sum_{i=1}^{k}f_{\mathrm{pti}},\;(1<k\leq z_{c})\;,$$

The total cumulative pitch deviation is

(8) $$F_{p}=\max(F_{\mathrm{pk}})-\min(F_{\mathrm{pk}})\;,$$

Note: $\left(z_{c}\right)$ denotes the number of cycloidal gear teeth. The individual pitch deviation $\left(f_{\mathrm{pti}}\right)$ reflects the uniformity of adjacent tooth spacings, while the total cumulative pitch deviation $\left(F_{p}\right)$ characterizes the overall rotational accuracy of the gear.

### 4.2. Tooth Profile Deviation Measurement

Through pixel equivalent calibration, light intensity compensation, and coordinate transformation, the coordinate points of the cycloidal gear tooth profile are converted from the pixel coordinate system to the physical coordinate system and then transformed into polar coordinates. By comparing the polar radii of each point, 29 approximate tooth roots were identified. Using these 29 approximate tooth roots, all scanned points were divided into 29 tooth profile point groups, with each group encompassing one complete single tooth profile.

**Figure 4:**
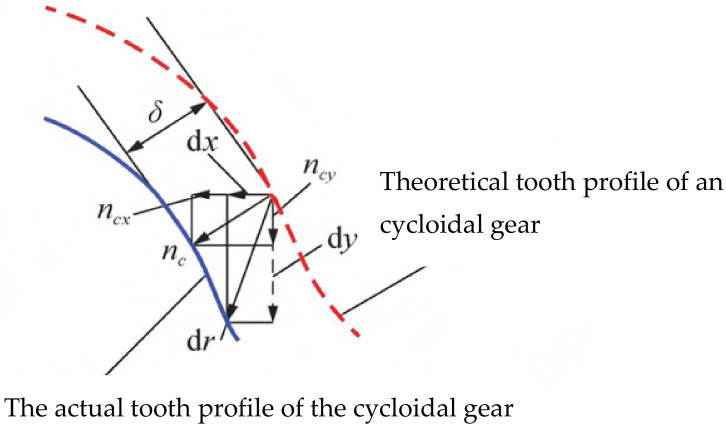
Calculation principle of tooth profile deviation.

The tooth profile deviation of a cycloidal gear refers to the normal distance between a complete tooth profile on the end plane and the theoretical profile [34]. Based on Equation (9), a theoretical tooth profile model is established. The theoretical coordinates are discretized into a finite set of points and converted to polar coordinates. Using the measured actual coordinate points as references, corresponding points on the theoretical profile are identified by matching polar angles. The deviation δ at each discrete point is calculated as the normal distance between the actual coordinates (mx, my) and the theoretical coordinates (x,y). The tooth profile deviation for a single tooth is denoted as Fα. Figure 4 illustrates the calculation principle of tooth profile deviation.

(9) $$\left\{\begin{array}{l} n_{cx}=\left(\cos\left((1-i^{H})\cdot \varphi \right)-K_{1}\cdot \cos(i^{H}\varphi )\right)\cdot s^{-1/2}\\ n_{cy}=\left(\sin\left((1-i^{H})\cdot \varphi \right)+K_{1}\cdot \sin(i^{H}\varphi )\right)\cdot s^{-1/2}\\ dx=x_{m}-x;\\ dy=y_{m}-y;\\ \delta =dx\cdot n_{cx}+dy\cdot n_{cy};\\ F_{\alpha }=\delta_{max}-\delta_{min} \end{array}\right.,$$

In the formula, $i_{H}$ denotes the transmission ratio coefficient; $K_{1}$ is the short-amplitude coefficient of the cycloidal tooth profile; s represents the normal vector normalization factor; φ is the polar angle; $n_{cx}$ and $n_{cy}$ are the horizontal and vertical components of the unit normal vector of the theoretical profile; $x_{m},y_{m}$ and x,y are the measured and theoretical coordinates, respectively; dx,dy are their coordinate differences; δ is the single-point normal deviation; $F_{\alpha }$ is the total tooth profile deviation of a single tooth, equal to the difference between the maximum and minimum values of δ.

## 5. Experiments and Analysis

### 5.1. Experiment Setup and Specimen Basic Parameters

Experimental tests were performed on three RV-6E cycloidal gears manufactured in the same batch. Table 2 presents the basic geometric parameters of the test specimens. A B&S EXPLORER coordinate measuring machine (CMM) with a maximum permissible error of 2 μm and a resolution of 0.1 μm was used as the reference instrument. The vision measurement system consisted of an MV-HS2000GM CMOS camera(Shaanxi Microvision Intelligent Manufacturing Co., Ltd., Xi’an, China), a BT-2380 dual telecentric lens, and an LED parallel backlight. All measurements were implemented in a thermostatically controlled laboratory at 20 ± 0.5 °C under constant illumination to reduce environmental interference.

### 5.2. System Calibration

The pixel equivalent is a fundamental parameter for vision measurement and strongly affects the overall detection accuracy [35]. For a vision system with a fixed hardware and working distance, the pixel equivalent remains constant and is defined as the ratio of physical dimensions to corresponding pixel dimensions [36]. Nevertheless, the limited dynamic range of image sensors easily causes local over-saturation or under-saturation under varying illumination, which induces directional deviations in edge positioning and interferes with pixel equivalent calibration. To solve this problem, a light intensity compensation coefficient was adopted during the calibration process to correct illumination-induced edge distortion, so as to guarantee the accuracy and repeatability of the calibration results.

A 7 × 7 dot-matrix calibration plate was used for pixel equivalent calibration. The plate contained 49 circular markers; each marker had a diameter of 2 mm, and the center-to-center spacing between adjacent markers was 4 mm, with an overall geometric error lower than 1 μm. To eliminate systematic errors caused by single shooting posture, we captured calibration images with the plate placed at three different rotation angles. The calibration plate was placed at the center of the camera’s field of view, and images were captured under a fixed light intensity. Subpixel edge detection and center fitting were implemented for nine circular markers in the central 3 × 3 region of the plate. Illumination variations would shift edges along their normal directions, while the center distance between markers remained stable. Accordingly, the pixel equivalent was calculated via the ratio of physical size to pixel size. The average pixel distance from the central marker to its eight surrounding markers was computed, and the final pixel equivalent of the vision system was determined as 21.7657 μm/pixel. Relevant studies have demonstrated that planar calibration plates can be applied to telecentric vision systems to simplify calibration workflows [37].

As described in the foregoing subpixel edge localization method (Section 2.2), the ruled-sigmoid model relies on normalized grayscale values within edge transition regions. Image over-saturation and under-saturation would distort grayscale distribution and produce systematic edge offsets [38]. To suppress this adverse effect, a light intensity compensation coefficient λ was introduced to revise normalized grayscale values before the logarithmic transformation in Equation (3).

The coefficient λ was calibrated using standard gauge blocks with a nominal thickness of 20 mm. The calibration was performed with λ∈[−0.3,0.3] at an interval of 0.01. When λ=0.13, the corresponding pixel distance of the gauge block was 918.871 pixels, and the measurement error reached the minimum value of −0.11 μm. After calibration, repeatability verification was carried out via ten consecutive measurements on the 20 mm gauge block. The standard deviation was calculated as 0.06 μm, which proved the reliable stability of the compensation coefficient. Gauge blocks of 10 mm, 30 mm, and 40 mm were further tested for validation. The mean absolute measurement error across all tests was less than 1 μm, which verified the feasibility of λ=0.13.

### 5.3. Single-Specimen Measurement and CMM Comparative Test

#### 5.3.1. Subpixel Edge Extraction and Tooth Profile Reconstruction

**Figure 5:**
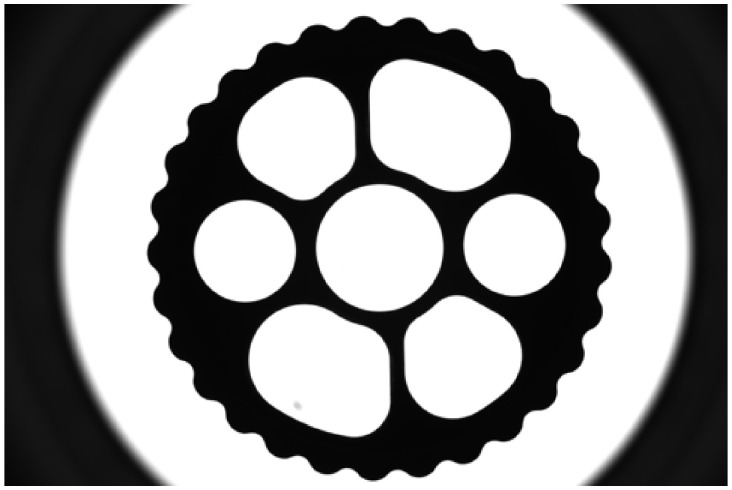
Cycloidal gear measurement image.

Image preprocessing was first conducted on the captured cycloidal gear image (see Figure 5), where Gaussian filtering was adopted to suppress background noise, and a dual-threshold segmentation method was used to extract the grayscale transition band of the gear tooth profile edge [13,14]. Subsequently, the proposed ruled-sigmoid subpixel algorithm was applied to achieve high-precision edge localization within the transition region [32].

**Figure 6:**
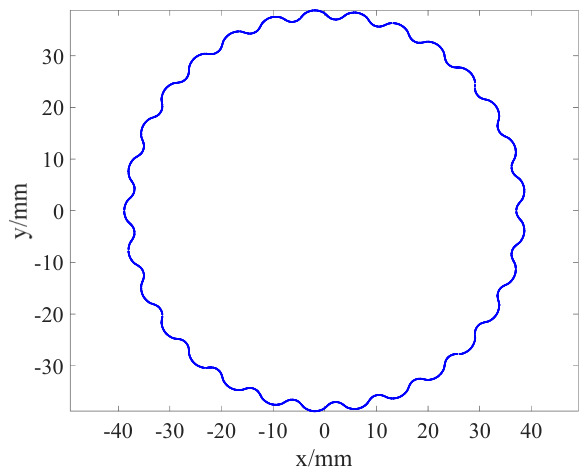
Subpixel edge of cycloidal tooth profile.

With the calibrated pixel equivalence coefficient and established coordinate transformation model, pixel-based subpixel edge coordinates in Figure 6 were converted into physical dimensions. Subsequently, discrete edge data were fitted via the NURBS algorithm to reconstruct continuous tooth profile curves [39]. Based on the theoretical tooth profile equation of cycloidal gear, computational models for tooth profile deviation and pitch deviation were established for subsequent quantitative evaluation [40].

#### 5.3.2. CMM Measurement Process and Measurement Point Planning

**Figure 7:**
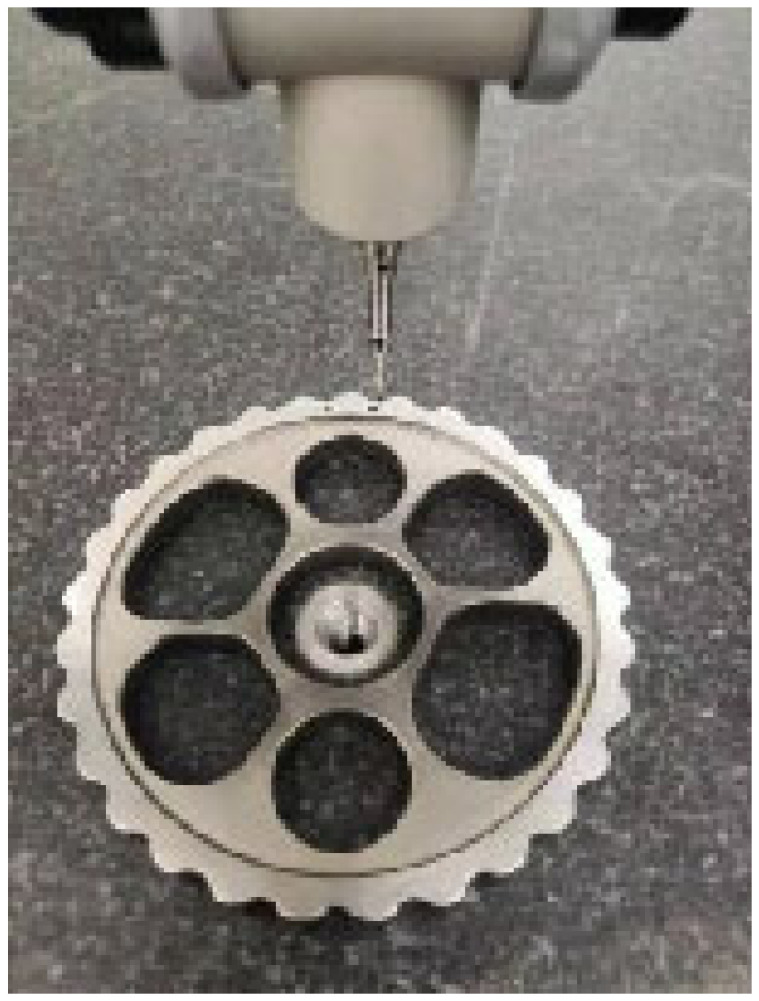
The measuring scene of the CMM.

A B&S EXPLORER coordinate measuring machine (CMM) was used to acquire reference tooth profile data for quantitative comparison. The instrument had a maximum permissible error of 2 μm and a resolution of 0.1 μm, and all measurement operations were implemented in accordance with ISO gear measurement specifications. The CMM and its supporting air supply system were powered on and preheated prior to testing for thermal stabilization, which eliminated the adverse effects of operational fluctuations on measurement results. The cycloidal gear specimen was thoroughly cleaned and rigidly mounted on the CMM workbench, as shown in Figure 7.

For the establishment of a unified measurement datum, four sampling points were acquired near the mid-height region of each crankshaft bore end face. The least-squares fitting algorithm was applied to calculate the geometric center of each bore. The midpoint of the line connecting the two bore centers was defined as the origin of the measurement coordinate system. The line linking the two bore centers was set as the positive *X*-axis, and its perpendicular horizontal direction was defined as the positive *Y*-axis. In accordance with the nominal CAD model of the gear, 22 sampling points were arranged counterclockwise on each tooth profile starting from the positive *X*-axis. The CMM probe moved automatically along the predefined trajectories to collect discrete profile coordinates and store the reference datasets.

Notably, the CMM adopted a sparse discrete sampling mode, while the vision system captured dense continuous contour data. Such differences in sampling strategies led to local point-to-point deviations between two sets of results. Relevant discussions are presented in the subsequent result analysis and GUM-based measurement uncertainty evaluation sections.

#### 5.3.3. Single-Sample Size and Tooth Profile Deviation Analysis

After the vision measurement and CMM reference data were fully collected, dimensional consistency and tooth profile characteristics of the tested cycloidal gear were analyzed. Table 3 summarizes the diameter results of the central hole and two crankshaft holes measured by the two methods, as well as the corresponding deviations. The maximum dimensional error between the two approaches was 8.0 μm for the central hole, while the deviations of the two crankshaft holes were only 3.9 μm and −0.9 μm, respectively. The above results demonstrated good agreement in dimensional measurement between the proposed vision system and the CMM.

**Figure 8:**
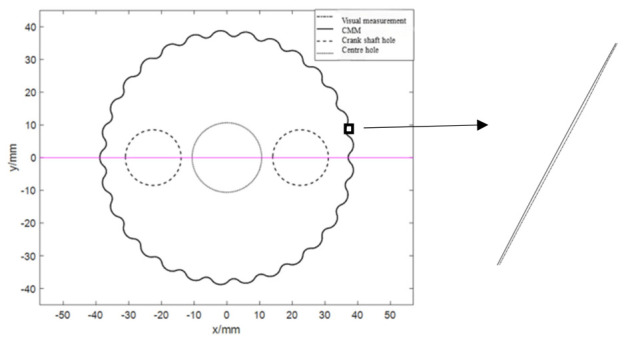
Comparison of cycloidal gear profile obtained by the two methods.

**Figure 9:**
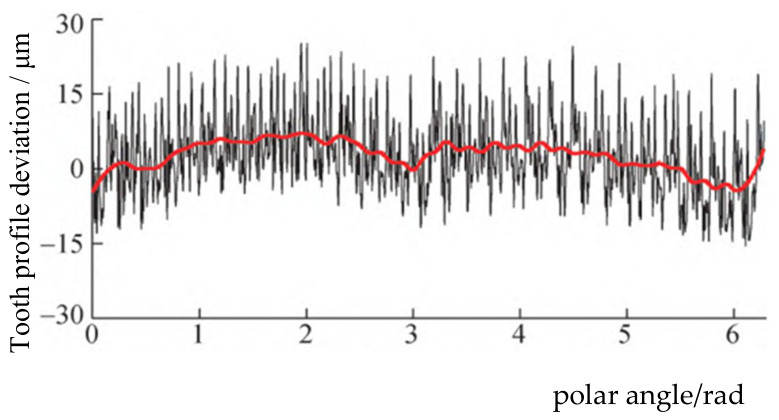
Deviation distribution of all tooth profiles.

The overall tooth profile curves obtained by the two measurement techniques were superimposed for visual comparison, as illustrated in Figure 8. The contour trends maintained high consistency across the full gear profile. Figure 9 shows the distribution of tooth profile deviation against the polar angle, with a deviation range from −4.5395 μm to 7.083 μm. This variation represents the inherent manufacturing deviation of the cycloidal gear itself.

**Figure 10:**
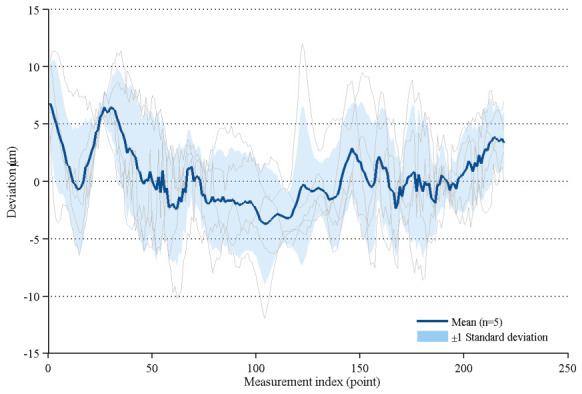
The profile differences obtained by the two measurement methods.

To further evaluate the profile difference between the two detection methods, five representative teeth (Nos. 1, 7, 13, 19, 25) were selected for comparative analysis, and the corresponding deviation curves are shown in Figure 10. Statistical results indicated that 93.83% of all sampling points had deviations within the range of ±10 μm, while the remaining 6.17% of points were identified as outliers with a maximum local deviation of 11.62 μm. These outliers were mainly distributed at the addendum and dedendum regions of the gear teeth.

The main causes of abnormal points included abrupt curvature at tooth tips and roots, tiny surface burrs on the workpiece, as well as the essential difference between sparse discrete sampling of the CMM and dense continuous contour extraction of the vision system. Mismatched feature collection positions also led to local deviations. It should be clarified that these outliers only reflected random sampling errors, rather than systematic errors of the vision measurement system.

**Figure 11:**
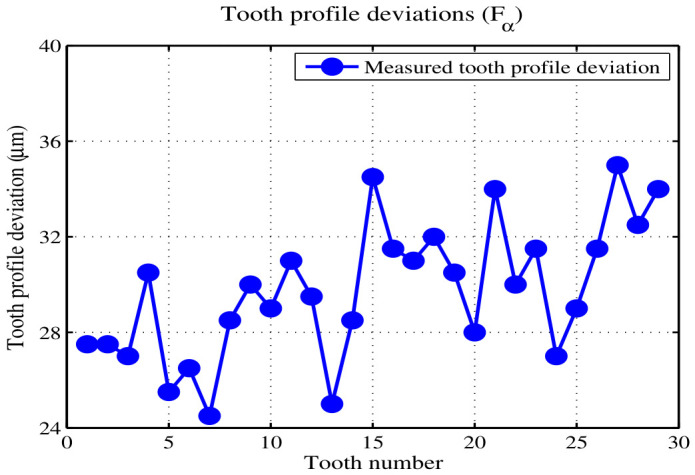
Statistical distribution of the overall tooth profile deviation $\left(F_{\alpha }\right)$ of the tested gear.

**Figure 12:**
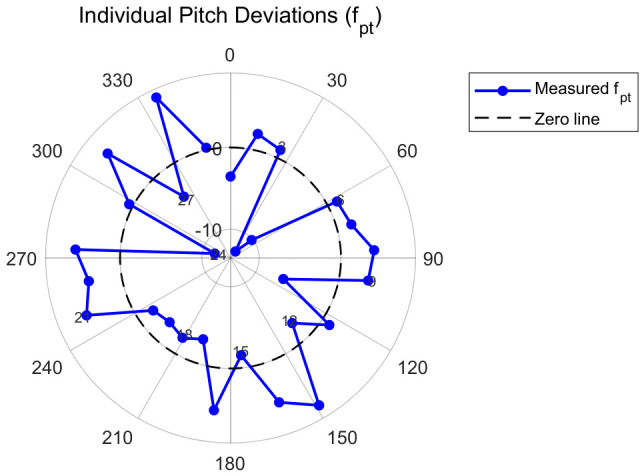
Distribution of individual pitch deviation $(f_{pt}).$

**Figure 13:**
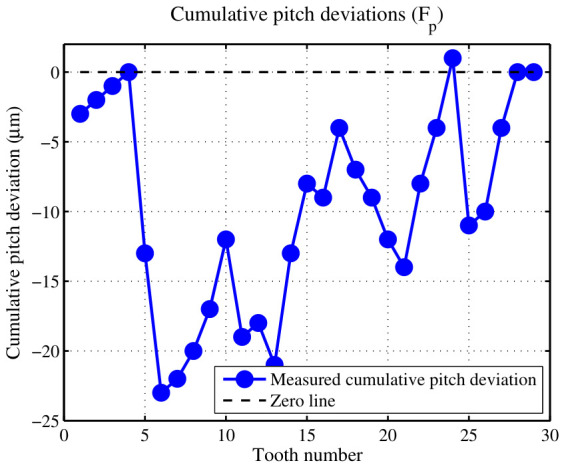
Distribution of cumulative pitch deviation.

Key evaluation indices of the tested single gear were calculated and recorded: the overall tooth profile deviation was 36.0570 μm, and the individual pitch deviation was 11.9121 μm. The distribution rules of tooth profile deviation, single pitch deviation and accumulated pitch deviation are presented in Figure 11, Figure 12, and Figure 13, respectively. The total inspection time for a single cycloidal gear by the vision system was approximately 13 s, which showed a prominent efficiency advantage compared with the traditional contact measurement mode of CMM.

### 5.4. Repeatability and Multi-Specimen Verification

Tests for repeatability and multi-specimen validation were conducted to comprehensively evaluate the stability and generalization performance of the proposed vision measurement system.

#### 5.4.1. Repeatability Test of Single Specimen

The No. 1 RV-6E cycloidal gear was selected for repeated measurements. Illumination, workpiece placement, and ambient temperature were all kept constant throughout the test, and ten repeated measurements were completed consecutively. Table 4 summarizes the mean value, sample standard deviation, and measurement repeatability of tooth profile deviation $F_{\alpha }$ and individual pitch deviation derived from the ten groups of test data.

Statistical results showed that the relative standard deviation (RSD) of $F_{\alpha }$ was 1.03%, and the RSD of individual pitch deviation was 2.44%. The corresponding repeatability values were 1.04 μm and 0.81 μm for the two indicators. The low RSD and repeatability values indicated that the system produced minor random errors and possessed excellent short-term measurement stability under fixed experimental conditions.

#### 5.4.2. Multi-Specimen Comparative Test

Two additional RV-6E cycloidal gears (No. 2 and No. 3) from the same production batch were tested under identical experimental conditions and operating procedures. Table 5 lists the measured, individual pitch deviation, and maximum deviation against CMM results for the three specimens. The values of each indicator fluctuated within the range of conventional manufacturing tolerances for batch-produced gears.

A one-way analysis of variance (ANOVA) was applied to the multi-specimen datasets. At the 95% confidence level, the calculated F-value was 1.29, and the corresponding *p*-value was 0.316. Since the *p*-value was greater than the significance level of 0.05, no statistically significant difference was observed among the measurement results of the three specimens. The results demonstrate that the vision method achieved stable performance and satisfactory universality for cycloidal gears of the same specification.

In this study, each specimen was measured only once, and the sample size was limited to three specimens, which represented a simplified ANOVA setup. The obtained results were valid for evaluating the practical performance of the system under typical industrial inspection scenarios. Further studies will adopt more specimens and repeated measurements per sample to improve statistical rigor.

### 5.5. GUM-Based Measurement Uncertainty Evaluation

The measurement uncertainty of the proposed vision system was evaluated in strict accordance with the ISO/IEC Guide 98-3 (GUM) [41]. This evaluation focused exclusively on the inherent error of the vision measurement system and excluded the manufacturing deviation of cycloidal gear workpieces. The total uncertainty was divided into Type A uncertainty for random errors and Type B uncertainty for systematic errors [42].

#### 5.5.1. Type A Standard Uncertainty

Type A uncertainty was calculated using the repeated measurement data presented in Table 4. The corresponding computational formula is as follows:

(10) $$u_{A}=\sqrt{\frac{1}{n(n-1)}\sum_{i=1}^{n}(x_{i}-\bar{x})}$$

where n=10 stands for the number of repeated measurements, $x_{i}$ denotes a single measured value, and $\bar{x}$ is the arithmetic mean of all measured results. According to the sample standard deviation s=0.37 μm, the calculated Type A standard uncertainty was $u_{A}\approx 0.117\;\mu m$.

#### 5.5.2. Type B Standard Uncertainty

Five independent systematic error sources were quantified. In accordance with GUM §4.3.7, uniform rectangular distributions were adopted for all Type B uncertainty components due to the absence of detailed probability distribution data for each error source. The half-interval of each error range and its specific derivation basis are explicitly described as follows:

1. Pixel equivalent calibration uncertainty: $(u_{B1}=1.85/\sqrt{3}$ μm). The half-interval value of 1.85 μm originates from the maximum residual deviation obtained after multiple repeated calibration experiments using the dot-matrix calibration plate.
2. Residual lens distortion uncertainty: $(u_{B2}=1.20/\sqrt{3}$ μm). The 1.20 μm half-interval is determined from the maximum residual error after polynomial distortion compensation for the dual telecentric lens.
3. Thermal drift uncertainty: $(u_{B3}=0.65/\sqrt{3}$ μm). This value corresponds to the maximum pixel offset converted from the allowable temperature fluctuation range of (20 ± 0.5 °C) in the constant-temperature laboratory environment.
4. Image grayscale noise uncertainty: $(u_{B4}=0.55/\sqrt{3}$ μm). The half-interval is extracted from the maximum grayscale fluctuation amplitude of static background images under stable backlight illumination.
5. CMM reference instrument uncertainty: $(u_{B5}=0.80/\sqrt{3}$ μm). This half-interval is converted from the maximum permissible error (MPE) index provided in the official specification of the B&S EXPLORER coordinate measuring machine.

All Type B components were mutually independent. The combined Type B standard uncertainty was calculated via the root-sum-square method:

(11) $$u_{B}=\sqrt{u_{B1}^{2}+u_{B2}^{2}+u_{B3}^{2}+u_{B4}^{2}+u_{B5}^{2}}$$

The combined Type B standard uncertainty was $u_{B}\approx 1.441$ μm.

#### 5.5.3. Combined and Expanded Uncertainty

Type A and Type B uncertainties were uncorrelated. The combined standard uncertainty was obtained as:

$$u_{c}=\sqrt{u_{A}^{2}+u_{B}^{2}}$$

Substituting the above results, the combined standard uncertainty was $u_{c}\approx 1.446$ μm.

For industrial precision measurement, the coverage factor k=2 was adopted, corresponding to a 95% confidence level. The expanded uncertainty was calculated by:

$$U=k\cdot u_{c}$$

The final expanded uncertainty of the vision measurement system was U≈2.891 μm.

#### 5.5.4. Result Discussion

The expanded uncertainty U=2.891 μm represents the intrinsic measurement error of the developed vision system. As discussed in previous sections, the maximum point-to-point deviation of 11.62 μm between the vision system and CMM was mainly caused by different sampling strategies and inconsistent feature extraction positions, rather than the inherent error of the vision system. Combined with the gear manufacturing deviation, the three types of errors were clearly distinguished in this work. The low expanded uncertainty fully proved that the proposed vision system satisfied the precision requirements for high-precision cycloidal gear inspection.

## 6. Conclusions and Prospects

### 6.1. Conclusions

1. A high-precision vision measurement system for cycloidal gears was designed and constructed in this paper. Adopting a bi-telecentric lens, a high-resolution CMOS camera, and parallel backlight illumination, combined with a precision motion and calibration scheme, the system enables non-contact, full-field, and high-efficiency detection of geometric parameters of tooth profiles.
2. A subpixel edge localization algorithm based on the ruled-sigmoid surface is proposed. The nonlinear grayscale surface is linearized via logarithmic transformation and solved by the least-squares method, which significantly reduces computational complexity while improving edge localization accuracy and noise robustness. A light intensity compensation mechanism is introduced to effectively suppress edge offset errors caused by image over-saturation or under-saturation.
3. A comprehensive error quantification model including pitch deviation and tooth profile deviation is established. Using a hybrid Cartesian-polar coordinate analytical method, synchronous evaluation of key geometric tolerances and polar-angle-dependent characteristics of tooth profile deviations is realized, providing a quantitative basis for cycloidal gear machining optimization and RV reducer transmission analysis. The results also reveal a clear polar-angle dependence of the profile deviation, which has not been explicitly reported in previous studies.
4. System calibration and comparative experiments show that the pixel equivalent was accurately calibrated and the light intensity compensation coefficient was effective. The edge localization accuracy reached 0.1 pixel, and the overall system positioning accuracy was better than 0.8 μm. Compared with a coordinate measuring machine (CMM), the profile deviation of the cycloidal gear was stably controlled at the 10 μm level, and the complete inspection cycle for a single workpiece was about 13 s, verifying the high precision, reliability, and efficiency of the proposed method.

### 6.2. Limitations of the Present Work

1. The imaging and calibration procedures of the current measurement system still rely on a stable laboratory environment, and its robustness against common industrial disturbances such as strong vibration, temperature and humidity fluctuations, as well as oil contamination or rust on workpiece surfaces, needs further verification and improvement. All existing measurement experiments were carried out under a fixed 20 ± 0.5 °C constant-temperature laboratory environment with stable backlight illumination; no interference simulation tests were set up in the current research design, which restricts the direct application of the system to on-site production lines.
2. The subpixel edge localization algorithm based on the ruled-sigmoid surface exhibited lower accuracy in regions with abrupt curvature changes, such as the addendum and dedendum of cycloidal gears, compared with the main tooth profile region. The parameter robustness in low-contrast and high-noise images also requires further optimization.
3. The error measurement model in this work is mainly established for standard geometric deviations (pitch and tooth profile deviations) and does not account for non-ideal factors affecting transmission performance, such as tooth surface micro-roughness and machining burrs, indicating that the engineering coverage of the model needs to be expanded.
4. The system has only been verified on cycloidal gears of fixed specifications. Only three workpieces from the same production batch were used for multi-specimen ANOVA statistical analysis, resulting in a small sample size that weakens the statistical reliability of the test results. Its generality for cycloidal gears with different modules, tooth widths, and modification forms, as well as its long-term stability in batch inspection scenarios, requires further validation.

### 6.3. Future Work

1. System Robustness and Engineering Improvement: Future work will introduce active vibration damping modules, constant temperature control, and anti-contamination illumination schemes, along with adaptive lighting and real-time calibration algorithms, to enhance the system’s stability and anti-interference capability under complex industrial conditions, and promote the transformation of the measurement system to online production inspection applications. A series of contrast experiments with vibration, temperature fluctuation, and workpiece surface pollutants will be specially designed in follow-up research to quantitatively evaluate the improvement effect of the above optimized schemes and make up for the lack of anti-interference verification in the current research design.
2. Algorithm Optimization and Generalization Enhancement: For regions with abrupt profile curvature and low-quality image scenarios, a hybrid edge localization model with higher robustness will be constructed by combining deep learning-based edge detection with traditional subpixel localization algorithms. The solving process of the ruled-sigmoid surface will be optimized with parallel computing and hardware acceleration to further improve inspection efficiency and enable real-time online measurement.
3. Expansion of the Error Model and Multi-Physics Coupling Analysis: Based on the existing geometric deviation model, non-ideal factors such as tooth surface roughness, burrs, and material elastic deformation will be introduced to establish a multi-factor coupled error prediction model. Combined with dynamic simulations, the influence of geometric errors on the transmission accuracy, noise, and service life of RV reducers will be analyzed, providing more comprehensive theoretical support for machining optimization and error tracing.
4. Multi-Specification Adaptation and Batch Inspection Applications: The measurement range of the system will be expanded, and an automatic adaptation algorithm for multi-specification cycloidal gears will be developed to enable parametric measurement of gears with different modules, tooth widths, and modifications. An automatic loading/unloading and data management platform will be built to form an integrated solution for batch inspection, data statistics, and quality traceability, promoting the large-scale application of the technology in the intelligent manufacturing of cycloidal reducers. More test workpieces from multiple production batches will be collected to expand the sample capacity, optimize the statistical reliability of ANOVA analysis and eliminate the limitation of small sample size in the original research design.

## Figures and Tables

**Table 1:** Performance comparison of different subpixel algorithms.

Algorithm	Positioning Error (Pixel)	Repeatability (Pixel)	RMSE (Pixel)	Time (ms)
Gaussian integral surface	0.15	0.05	0.17	28.6
Zernike moment	0.23	0.06	0.26	19.3
Proposed ruled-sigmoid	0.08	0.01	0.09	12.7

**Table 2:** Basic parameters of the RV-6E cycloidal gear.

Name	Symbol	Value
Number of cycloid gear teeth	$z_{c}$	29
Number of pin teeth	$z_{p}$	30
Radius of pin distribution circle	$r_{p}$	39.81 mm
Pin radius	$r_{rp}$	1.845 mm
Eccentricity	a	0.885 mm

**Table 3:** The diameter of the center hole and crank shaft hole detection/mm.

Test Object	CMM	Vision	Error
Center hole	21.2426	21.2496	0.0080
Crank shaft hole1	17.0029	17.0068	0.0039
Crank shaft hole2	17.0030	17.0021	−0.0009

**Table 4:** Repeatability of repeated measurements (No. 1 specimen, n =10).

Item	Mean/μm	Std/μm	Repeatability/μm
Fα	36.05	0.37	1.04
fpt	11.90	0.29	0.81

**Table 5:** Measurement results of the multi-specimen tests.

Specimen	Fα/μm	fpt/μm	Max. Deviation vs. CMM/μm
No. 1	36.06	11.91	11.62
No. 2	34.82	11.35	11.04
No. 3	35.47	11.68	11.31

## Data Availability

No publicly archived datasets were produced during this research. All measurement raw data involved proprietary equipment parameters and are retained privately within the research group. Data access may be granted from the corresponding author after submitting a formal reasonable application.
